# Developmental authenticity - underlying dynamics of inner work processes

**DOI:** 10.3389/fpsyg.2024.1231484

**Published:** 2024-06-12

**Authors:** Kerstin M. Liesenfeld, Sabine Lebedinski, Anna Katharina Parks, Olaf Dammann

**Affiliations:** ^1^Liesenfeld Research Institute, Boston, MA, United States; ^2^Department of Public Health and Community Medicine, Tufts University School of Medicine, Boston, MA, United States; ^3^Department of Gynecology and Obstetrics, Hannover Medical School, Hannover, Germany

**Keywords:** personality development, authenticity, growth mindset, new work, inner work

## Abstract

An emerging body of research attends to grasp the concept of authenticity. Nonetheless, a view on the developmental part with its underlying facets of *Inner Work* processes, is rare. In this paper, we aim to take a deeper look into the dynamics of inner work processes, that define certain authenticity developmental stages. Building upon our recently proposed “4C-view” of authenticity, we approach this developmental perspective from two different angles: from a *process characteristic* angle and a *developmental level* angle. Using vignettes of authentic client personality coaching processes, we propose that the interwoven dynamic between those two aspects yields several combinations of maturity levels within the individual. Continuity as an overarching concept thus contains various developmental authenticity stages that could be identified through different markers and vary in particular contexts.

## Introduction

Authenticity has gained traction in personal psychology and leadership literature. Specifically, *New Work* approaches ask for the so-called *Inner Work* or continuous *self-development* to generate successful sustainable change and transformation processes (Laloux, 2014; Scharmer, 2014). Scholars and practitioners agree that authentic leadership development strategies are required to face new challenges and to realize desirable outcomes especially in turbulent times (Seligman, 2002; George, 2003; Luthans and Avolio, 2003). Nonetheless, most leadership theories fail to investigate the essential processes that focus on the developmental aspect within the individual and the inner immunity that undermine personal development (Kegan and Lahey, 2009). Instead of testing post-hoc analyses and concepts we aim to highlight dynamic developmental processes within an individual or group in context.

The concept of authenticity can be viewed from various angles: Toper et al. (2022) acknowledged authenticity as a two-factorial construct, an alignment with the self (inner aspect) and no need of endorsement from the outside (outer aspect). In his view the inner aspect refers to the ability to notice one’s true self and to attend to internal processes, whereas the outer aspect shows the independence of the person regarding the approval of others (Toper et al., 2022). Cartwright et al. (2023) supports this definition and describes an authentic person as someone who lives and expresses oneself in congruence by its desires, ideals, and motives. Reznichenko et al. (2021) considers authenticity from two angles—as a person-centered approach which aligns with the definitions of Toper et al. (2022) and Cartwright et al. (2023) and in addition, as a holistic phenomenon which places more emphasis on an authentic relation between an individual and its outer world consisting of relationships and social norms (personal authenticity). All these approaches insist on a congruence within oneself and surroundings. Guenther et al. (2023) challenges this view by examining that self-enhancement and authenticity concur with each other—people think of themselves as authentic if they think highly of themselves. This second aspect seems to contradict the assumption of authenticity in the sense of being true to oneself since authenticity would also include negative traits, beliefs, and behavior.

Apparently, the term authenticity lacks a distinct definition which has also been described by Newman (2019). This led Lehman et al. (2019) to formulate a rather nuanced one. Lehman et al. (2019) offer three aspects of authenticity within an individual or group. The authors proposed reference groups that each provide a different perspective for the evaluation of authenticity, but still include the aspect of being true in relation to a referent (Jongman-Sereno and Leary, 2018; Lehman et al., 2019; Sedikides et al., 2019). Authenticity can be viewed in terms of *consistency*, *conformity*, and *connection*. In this instance an entity can be a person or a group for the first two aspects and mainly an object for the third aspect. First, it is the entity’s consistency with their internal values and beliefs which presents itself through their behavior. The sources of values and beliefs can be found in longstanding experience-based (through the interaction with the context) personal convictions that eventually might serve as drivers for authentic identity formation. Second, Lehman and colleagues describe authenticity as the extent of an entity’s conformity to a claimed social category. Third, it is an entity’s connection to a moment in time or a specific place.

In addition to Lehman’s et al. (2019) concept we have suggested that an entity can be authentic in congruence with those three different aspects. Thus, we added a fourth dimension, *continuity*, to the 3C-model of Lehman et al. (2019), essentially proposing a 4C-model of authenticity (Dammann et al., 2021) that includes different perspectives on authenticity (Reznichenko et al., 2021; Toper et al., 2022; Cartwright et al., 2023). The continuity aspect gives rise to the idea we have come to call *Developmental Authenticity*, which can be described along two axes, *process characteristics and developmental level*. *Process characteristics* include (1) *Ongoing Self-Assessment*, which requires critical reflection to evolve, (2) *Dynamic Change*, that we all undergo regularly through developmental challenges, (3) *Breakage Points*, meaning critical life-incidents that have a vital impact on personal growth, and (4) *Never-Ending Story*, referring to either recurring behavioral and perception patterns or the never-ending development of humankind. *Developmental level* describes a non-hierarchical, dynamic situational state of development and refers to (1) *fragmentation*, (2) *partial integration* and (3) *holistic integration*.

## Theoretical framework: Developmental Authenticity in light of dynamic theories of personality

Personal development and growth are not static nor linear processes but develop in various strands and in different dynamic contexts (Fischer, 1980). Furthermore, static concepts like for example Big Five and Hexaco, support misconceptions about personality which include the idea that personality is fixed, decontextualized and binary (Jach et al., 2023). Meanwhile various theories have emerged, that try to embed the dynamic aspects of personality (see Hecht et al., 2023) and therefore contain stable (trait) as well as variable (state) aspects. These theories allow for describing people in general personality style terms (e.g., as extraverts or introverts) and also explain how momentary behavior manifests in daily life.

There are numerous personality theories that incorporate implicitly or explicitly dynamic aspects of personality and historically stem from the person-situation-debate. As one solution to this debate, Mischel and Shoda (1995) suggested the Cognitive Affective Personality System (Shoda and Smith, 2004; Dingess and Wilt, 2020), that refers to behavior as a result of situational cues and not only dependent on traits and is best described by if-then patterns (Hecht et al., 2023). In addition, the Whole Trait Theory (WTT) (Fleeson, 2001; Fleeson and Jayawickreme, 2015) builds on and extends the CAPS model by differentiating between descriptive (density-distribution of behavior) and explanatory (momentary behavior as a result of situation and action) aspects of personality (Hecht et al., 2023).

Newer theories, for example the Cybernetic Big Five Theory (CBFT; DeYoung, 2014) assume that people are human cybernetic systems who show behavior in relation to their goals or references which are then examined and possibly adjusted through feedback processes. In contrast to our continuous dynamic developmental approach, this theory is concerned with type related psychological individual differences and builds on a mechanistic operational cycle of five stages of goal activation, action selection, action, outcome interpretation and goal comparison (DeYoung, 2014). Another more recent dynamic personality theory, the Cues-Tendency Action Model (CTA model) by Revelle and Condon (2015) was built on the Dynamics of Action Model by Atkinson and Birch (1970) and is also more concerned with stable individual differences regarding dynamics of primary response approach and avoidance behavior. The CTA model theory highlights behavioral tendencies regarding action-, respectively inhibition-forces within or between individuals, respectively groups and individuals. Consequently, in line with personality style related approaches, CTA model theory focuses less on overarching developmental and personal growth aspects.

Eventually, dynamic models of personality development, specifically to be found in the neurofunctional design approach of Kuhl’s theory, the dynamic context-differentiated theory of Fischer’s and the life-long-learning approach of Erikson’s theory are more suitable to discuss our idea of developmental authenticity since those three theories include the concept of continuous non-linear development, which we refer to as *continuity* in our proposal of the Developmental Authenticity theory.

### Kuhl’s Personality System Interaction Theory

In his Personality System Interaction (PSI) Theory Kuhl (2001) describes growth and personal development through a dynamic interaction between psychological systems. The basis for this functional approach can be found in one of the most complex and functionally structured theories on personality by Carl Gustav Jung (1875–1961). Like Jung (1946), Kuhl sees the origins of behavior and development less in mental factors, such as habit-forming, thought constructs or intentions, but more in functional-analytical terms. For example, the functionality of one or several underlying systems is supposed to play a crucial role. Across several dimensions, personality development includes both functional dispositions and effective processes.

Essentially, PSI has four different cognitive macro-systems (*intuitive behavior control, object recognition system, intention memory, and extension memory*) that are involved in thinking, emotional, and action processes, while also interacting with one another. Their function and interaction are modulated by moods, i.e., by positive and negative emotions. In turn, these emotions are linked to the individual cognitive systems in various ways and promote or inhibit their respective functionality. The interaction between the four systems occurs in the form of co-operation between two elementary lower cognitive systems, i.e., intuitive behavioral control and object recognition and two higher cognitive systems, intention memory and the extension memory. Moreover, two system pairs emerge when intuitive behavioral control is paired with intention memory, and object recognition is paired with extension memory (Kuhl, 2001). While the first pair controls implementation energy for goals and their achievement (*action axis*), the second promotes learning from experience (*experience axis*).

The interaction between those four systems will naturally be challenged by dynamic changes over the entire course of human development. Specifically, the experience axis provides one of the key prerequisites for developmental authenticity. Kuhl’s *second modulation assumption* is devoted to the dynamic interaction between the elements of the experience axis (Kuhl, 2001).

The second modulation assumption postulates that by activating the object recognition system negative emotions impede or even block access to highly inferential systems of feeling (EM). Individuals on a fragmented level are not able to regulate the negative emotions and integrate their experiences into the extension memory. This tends to provoke situation(state)-orientation rather than self-access, thereby undermining developmental authenticity (Kuhl, 2001). Other individuals are on their way to integrate information from the object recognition system into the extension memory (partial integration). The ability to keep the two systems in equilibrium and letting both systems inform each other without blocking either one is a sign of holistic integration capabilities (Fischer, 1980; Kuhl, 2001).

Developmental Authenticity thus refers to the successful integration of single detached object representations (object recognition system) with holistic perception (extension memory) while keeping the two in equilibrium. The question arises, what is needed by the individual or their caregivers/role models, to support Developmental Authenticity, i.e., to scaffold the developmental journey from fragmented perception to holistically integrated perception. Apart from providing a nurturing environment, an adequate response during phases of heightened dynamic change, in particular during critical life incidents, appears to be of crucial importance (Liesenfeld, 2014; Baumann and Kuhl, 2021).

### Fischer’s Dynamic-Skill-Theory

Fischer (1980) offers a concept of skill development that works on the assumption of a fractionated development of minds. Minds develop in fractionated strands of a web that over time can potentially be integrated (Ayoub and Fischer, 2006). The theory conceptualizes four characteristic levels of development: first, *a natural split into positive-negative bias*; second, a *primitive level of integration* expressed in the ability to shift between positive and negative; third, *partial integration through Representational Mapping*; and fourth, *strong integration through Abstract Mapping*.

The first level describes the natural form of perception and information processing, divided into good and bad (natural splitting). The second level describes the “primitive” level of integration and is referred to as *Single Representations*—where a person is able to shift between positive and negative bias. In his research Fischer investigated children’s ability to retell a story of multiple dolls’ interactions. At the first level, they are able to grasp the concept of either being mean or being kind. This first natural split into positive-negative bias as well as the ability to shift between negative and positive, we refer to as the developmental level of fragmentation.

In Fischer’s Dynamic Skill Theory (DST), caregivers play an important role in modeling different responses to certain dynamics. The more children, especially during stressful times, are provided with an external regulation source through understanding, comforting, and giving support, the more they will be able to later downregulate negative affective states on their own. Consequently, they are more capable to enter skill levels of abstractions where they have access to various responses within dynamic situations instead of being caught on single representation levels of development, where they can only access a dichotomous level of understanding, expressed in good versus bad judgements/behavior (Fischer, 1980).

Fischer’s third level, the Representational Mapping describes the *combination of opposite categories* where different concepts are combined into one setting. For example, *Mean and Nice actions* are combined in one setting. During this part of cognitive development, children can observe and recount a person reacting in different ways to the same action. This level allows for first abstractions of objects, incidents, and people as untouchable concepts. The individual is able to realize that intentions matter more than actions. On this third level personality-relevant characteristics can be perceived, like “my father is emotional” or “my father is rational.” We follow Fischer here and infer our concept of partial integration from this level of development as each person goes through multiple stages of development, similar to a child during learning.

The fourth level of Abstract Mappings in Fischer’s Dynamic Skill Theory is characterized by strong integration capabilities. At this developmental level the individual is able to identify certain personality characteristics as harmonic or not and therefore as a sign of a special relationship. Strong integration allows for the understanding of moral principles of justice through integration of different kinds of relations between intention and responsibility. On this level the individual shows ambiguity tolerance, i.e., the ability to integrate contradictory perceptions into one holistic system, for example the relationship of parents or friendships that changes over time. On this strong integration level, skill development can be understood as a form of differentiation of already existing patterns that need to be further developed to function effectively. In our approach we refer to this developmental level as holistic integration.

### Erikson’s stages theory

Erik Erikson ranks as one of the so-called Freudian ego-psychologists and, like Freud, takes different stages of development into account in his theory. However, in his considerations he goes beyond adolescence into advanced adulthood, so that his theory covers a total of eight stages of personal development over an entire lifetime. Erikson (1959) places his focus less purely on instinctive urges or the subconscious, but also observes an additional psycho-social and psycho-historical component.

Although it is not explicitly stated how each individual stage can be mastered practically on the behavioral plane, the theory provides an overview of the personality competences to be developed in each stage. A closer look at the eight stages shows that his model seems to go beyond a mere ego-consideration, as the development tasks outlined in the different stages can be attributed less to an explicit, sequentially, and analytically working ego alone. On the contrary, the identity-forming conflicts outlined indicate an expanded perspective on personality also taking implicit aspects of the self into account.

Moreover, his consideration of development proceeds from so-called epigenetic stages, i.e., from a sequence of steps in which coping with one development task and learning in one stage act as the foundations for the next stage (cf. Erikson, 1959). His theory postulates that every development task may be linked to conflicts and crises and that sometimes no complete mastery takes place in one stage, but that an as comprehensive as possible coping with the development task in one stage facilitates entry to the next stage. Erikson’s theory further specifies that each new stage in a person’s life requires assessment of the previous stage to integrate a part of the new stage.

The stages can be described as follows: The first stage of development (*trust* vs. *distrust*) begins in the first year of life. It is crucial for building up primordial trust vs. primordial distrust. The formation of this primordial trust takes place through prompt, appropriate and loving responses to needs, which contribute to the experience of bonding security (see also Bowlby, 1951; Ainsworth and Bell, 1970). The adequate response from first caregivers to children while growing and developing is a main aspect of the first four developmental stages.

These responses include encouragement of a child’s autonomy (stage two—*autonomy* vs. *shame*), supporting the child while it develops first moral ideas (stage three—*initiative* vs. *guilt*) as well in its first setbacks or successes as an individual who acquires different roles in new contexts than previously learned (stage four—*diligence* vs. *inferiority*). The accomplishment of these four stages from the ages of one to twelve allows a further differentiation of personal identity from age 12 to 20 (stage five—*identity* vs. *role confusion*). It eases the integration of contradictions regarding the individual’s self (Erikson, 1968). This helps an adult to admit genuine personal intimacy, without abandoning a part of them (stage six—*intimacy* vs. *isolation*).

After acquiring knowledge and experience an individual starts to generously pass on experience and personal knowledge to younger generations (stage seven—*generativity* vs. *stagnation*). If all stages have been completed successfully, the last stage is defined by gaining wisdom (stage eight—*self-integrity* vs. *despair*). Going through each stage allows an individual to enhance emotional integration and this leads to an improved ability to differentiate perceptions of emotions.

In theory, the stages of Erikson mirror a perfect pathway of a psychologically and physiologically healthy as well as a socially well-off (e.g., loving, nurturing environment) individual. Nevertheless, this pathway is not carved in stone. Each stage can be completed at different times in one’s life and allows one to develop the abilities to integrate emotions. Nevertheless, it will be comprehensible that healthy experiences in the first stage facilitate coping successfully with the second stage (cf. Hazen and Durrett, 1982). With greater primordial trust as a foundation, children can explore their environment more uninhibitedly and free of anxiety to gain new experiences. For those who could not develop primordial trust or autonomy, it might be more challenging, but not impossible to enhance one’s own emotional integration in a lifetime by pursuing the stages in a different order and at one’s own pace.

Erikson’s theory also supports the various developmental levels within the dynamic development of the eight stages. His stage one symbolizes fragmentation whenever primordial trust could not be built as a foundation. The individual has the chance to partially integrate through regulating personal setbacks retrospectively over the course of various developmental stages (stage two to stage six). Holistic integration in Erikson’s theory shows in stages seven and eight, where the individual is generously passing on knowledge and experience (seventh stage) and gaining wisdom (eighth stage).

## Developmental Authenticity: a proposal

Within the framework of New Work, Inner Work governs self-self as well as self-other reflections and transformation processes in order to align necessary external change processes with the individual’s or entity’s inner mapping. Inner work processes thus will confront people with non-arbitrary interactive dynamics for example through the shift of values and beliefs, that eventually cause challenges to the self-concept, identity formation and eventually the self as a dynamic system. As identity is a core aspect of the self and located at the major crossroads of individual and social processes, the identity challenge will emerge on one hand from the concept of individuality—the person experiences himself or herself as a unique person—and on the other hand through a specific sense of belonging linked to the social context and recognized by the subject (Zacares and Iborra, 2015). The development of an identity and an authentic self is a complex, multifaceted growth process that is based on a series of interrelated conscious as well as unconscious developmental unfolding. Within these unfolding various self-representations will feed the identity, self-concept, and consequently self-formation through a continuous interaction between the person and the context, so that the self allows each person a privileged access to his or her own thoughts, feelings, and sensations (Baumeister, 1987).

**Figure 1:**
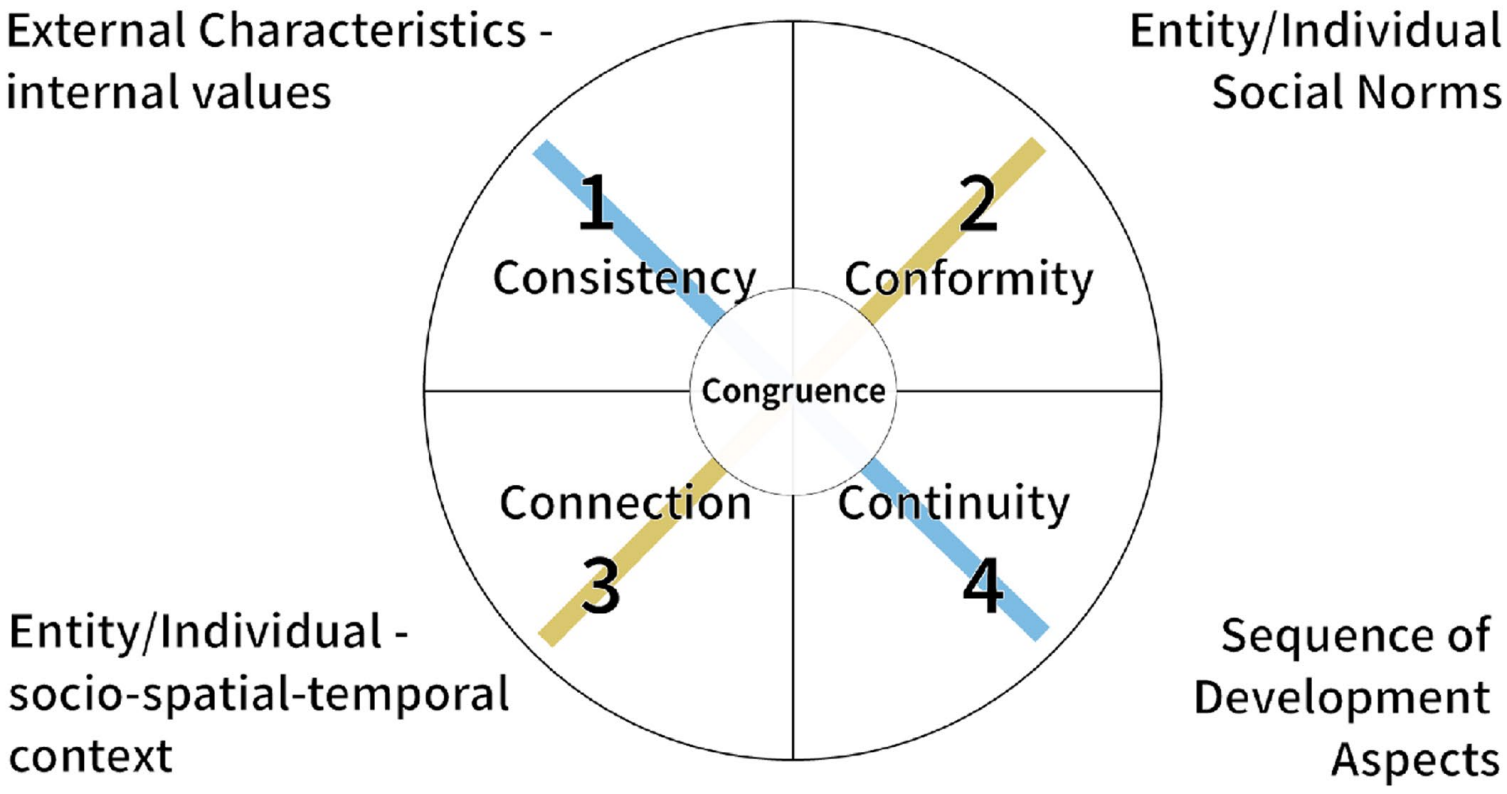
Congruence as essence of authenticity.

In this vein, self-concepts rather refer to the totality of inferences a person has made about him- or herself. This includes personality traits as well as an understanding of one’s social roles and relationships. In contrast, identity will more likely be subject to attribution from the context, created for and superimposed on the self (Baumeister, 1987). Only with a dynamic intra- and interindividual balance between the entity and the context, change and transformation processes can be successful (Scharmer, 2014). With our proposal of Developmental Authenticity, we aim to contribute to the inner work, that leads to successful transformation processes. By embracing the individual perception of authenticity and endorsing a complex view on natural growth dynamics we facilitate the maturation of the authentic self (see Figure 1).

**Figure 2:**
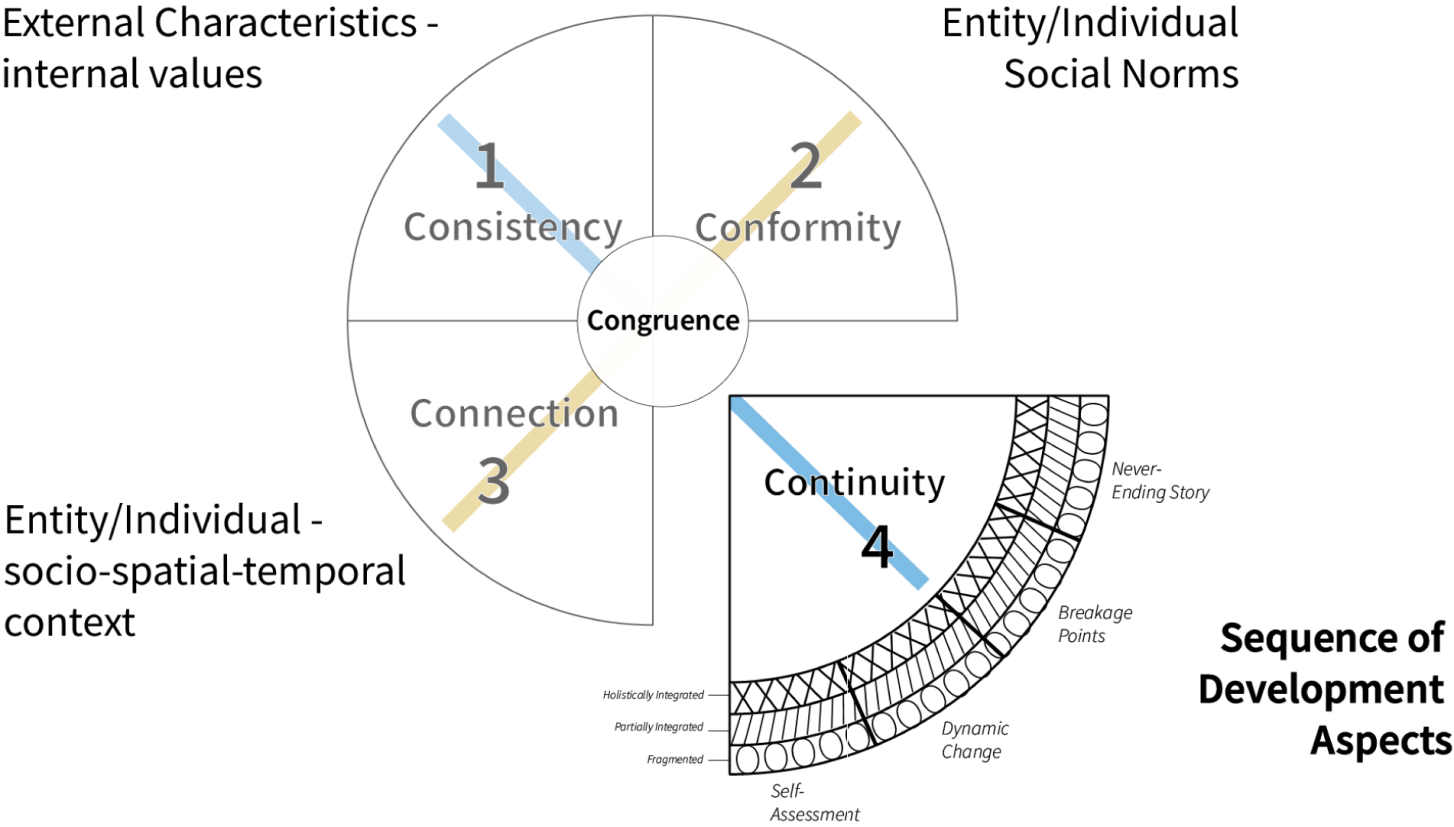
Sequence of development aspects—continuity.

As we pointed out in our recent article the essence of authenticity is in our view *congruence* (Dammann et al., 2021). We see congruence as a specific equilibrium of the self, regarding self, others, and relevant social norms. This equilibrium cannot be shifted without any developmental impact, in this sense, it requires continuous work and the willingness to change. An individual has to reflect whether these changes lead to interruptions of authenticity, e.g., on emotional or psychological levels (MacKenzie et al., 2007). The ability to evaluate one’s authenticity requires the development of cognitive abilities and fades out with age, e.g., due to dementia (see Figure 2).

We derive the concept of Developmental Authenticity from the fourth C, authenticity as continuity and characterize it as an inner sustainable development of one’s characteristics of an individual self (core) over a lifetime. Thus, the concept of authenticity proposed in this article is concerned with a form of self-development that comes close to the underlying inner developmental schema, i.e., the true nature of the person, which Kuhl calls “proto-self” (cf. Damasio, 2002; Kuhl, 2010). Or: and Dostojewski (1821–1881) referred to in his quote “to love somebody means to see him as God meant him to be”. The prerequisite for this kind of sustainable development of the true nature of the person is a “self-regulation-mode” that is able to assimilate the current emotional and perceptual world in the sense of the current goal through contact with existing values and schemata, from previous interactions with other people, one’s own feelings and habitual interpretations (“contact orientation”). A healthy developed self-regulation mode fosters a high degree of stability (self), security and social embedding (meaning) within the context, so consequently a stabile identity (Kuhl, 2001). As growth cycles are non-linear, authentic development might also entail phases of “loss of identity” through regressive developmental periods. Those regressive periods show an uncoordinated juxtaposition of processes controlling experience and action, resulting in inconsistent experience and action (Kuhl, 2010). In our *authenticity as continuity* approach, we consider these regressive periods also part of the authentic development whenever people are able to comprehend, integrate, and embrace the necessary associated transformations within themselves (Krause, 2017).

As Developmental Authenticity implies continuous change processes within a highly complex system, the question arises, how a certain state (as one aspect of the developmental journey) of *authenticity in a person* could be defined. To approach this question, we will borrow from the theory of social welfare economics, that describes *Pareto-optimal* social situations in which it is not possible to increase the welfare of one individual through a reallocation of resources without simultaneously decreasing that of another individual (see Arrow and Debreu, 1954; McKenzie, 1954; Nikaido, 1956; Negishi, 1960; Barnett, 1973).

In a similar vein, we consider a person as authentic (individual congruence), when on every level of development, it will not be possible to shift stronger towards one dimension (self-self, self-others, self-social norms) without at the same time losing the inner connection to the equilibrium of another dimension. This dynamic balance of inner constructs like values and external influences such as social norms and relations allow a person to experience a high degree of identity (Kuhl, 2001).

- Self-self (internal values—external characteristics).
- Self-developmental status (in comparison to the community).
- Self-others (social norms).
- Self-context (social position).

In what follows we will focus on self-self and self-other dynamics in the context of process characteristics (Table 1) and developmental level (Table 2). The below defined aspects lay the foundation for a matrix of Developmental Authenticity. The characteristics of continuity can be aligned with aspects of theories of individual development and thus create the basis for our theoretical approach to Developmental Authenticity (Table 3). The developmental aspect of authenticity as well as the development of individuals range from gradual development over time (Piaget, 1950; Kohlberg, 1969) to nonlinear dynamics (Fischer, 1980; Koole and Kuhl, 2003), where certain dynamics continuously challenge static concepts, and to authenticity as a skill that requires “authenticity work” (Peterson, 2005, p. 1086). On these grounds, we offer a proposal for *where* in the process of development the individual is at a certain point in time and whether their development is congruent to social norms and values or the development of the community.

**Table 1:** Definition of process characteristics.

Process characteristics	Definition
Ongoing Self-Assessment	Continuous re-evaluation of self, regarding own development, internal values (self-self), external requirements and self-others *Marker: subjective well-being and aiming to hold the individual perception of authenticity*
Dynamic Change	Ongoing evaluation/re-evaluation between the self-self- and self-others-dimensions. Inner fight between authenticity and inauthenticity *Marker: Growth cycles of the brain and concomitant dynamic development of self*
Breakage Points	Critical life incidents and/or growth cycles disrupting authenticity Less controllable changes within the self-self-dimension *Marker: Individual level of developmental status/short- term regression and long-term integration with personal growth*
Never-Ending-Story	Perception of various beginning points Discovering transgenerational never-ending-story patterns *Marker: Shift from fragmented personality with reactive perception/behavior into integrated personality with responsive perception/behavior*

**Table 2:** Definition of developmental levels.

Developmental levels	Definition
Fragmentation	Information processing and perception is fragmented as single representations Inner categorization scheme is binary “black or white,” “good or bad,” “either or” *Marker: Strong categorization, no holistic perspective taking, splitting of concepts and emotions*
Partial integration	Information processing and perception in mapping of representations. More differentiated perspectives on representational mapping Still fragmented single representations *Marker: More differentiated information processing that allows to integrate contradictions to a certain extent*
Holistic integration	Ability to holistically process information Moving from subject to object perspective on a holistic level Ability to show empathy Finding creative solutions as a spectrum of possibilities Self wins over the ego *Marker: holistic perception of events and transformative processing of information*

**Table 3:** Developmental authenticity mapping: process characteristics at certain developmental levels.

	Fragmented	Partially integrated	Holistically integrated
Ongoing Self-Assessment	Learning in single representations; passive dissociation	Integration of adverse events; increased self-awareness and self-assessment	Continuous evaluation and differentiation of self; ambiguity tolerance
Dynamic Change	Re-evaluation of change impossible	Partial ability to re-evaluate authentic and inauthentic aspects of self	Ability to re-evaluate authentic and inauthentic aspects of self
Breakage Points	Critical life incidents negatively affect personal growth	Partial personal growth; conscious awareness of feelings, cognition, experiences	Self-confrontation and integration facilitate personal growth
Never-Ending-Story	Stagnation of personal growth over life-course^a^	Increased conscious control over behavior and perception enables personal growth^a^	Personal growth and authentic development contribute to growth-mindset^a^

a All three levels of Never-Ending-Story potentially entail transgenerational growth effects, i.e., unconscious behavior and perception patterns, that has been transferred (epi)-genetically through generations.

As depicted in Table 1, Ongoing Self-Assessment refers to constant reevaluation of one‘s self since everything in life is bound to change. Dynamic Change refers to the ongoing evaluation and re-evaluation of a person’s ever-changing characteristics with the question in mind whether they fit their current perception of authenticity. Breakage Points refer to times in someone’s life where perceived authenticity is disrupted, possibly due to critical life incidents. Never-Ending-Story refers to the assessment of a beginning and an endpoint, where authenticity is or is not perceived (Dammann et al., 2021).

These process characteristics are a part of the continuous development of authenticity. A person may find themselves going through each state several times throughout their life in various capacities. Individual processes will be characterized by these four aspects throughout their life since dynamic change and breakage points are inevitable. The ability to self-assess is crucial to understand self-self and self-other relations. The Never-Ending Story aspect describes the phenomenon of a continuous pattern that we have found.

Our approach has its roots in various above mentioned developmental and learning theories for example Erikson (1959), Fischer (1980), and Kuhl (2001), each of whom have touched on parts of process characteristics that have been combined and defined in Table 1. These process characteristics alone would not suffice to explain the development of a person as the process may be repeated in cycles several times throughout a person’s life. During each cycle, the process may be experienced differently depending on the developmental level of an individual, such as fragmentation, partial integration, and holistic integration (Table 2).

*Fragmentation* describes the first developmental level where an individual processes information through single representations and dichotomous biases. *Partial integration* covers developmental levels where individuals can access broader and contradictory perspectives next to single representations. *Holistic integration* represents the individual’s ability to fully grasp contradictory perspectives and map them into complex representations.

In Table 3 we map *process characteristics* with *developmental levels* in a matrix. The matrix is further illustrated through selected vignettes of individual client cases. Each case will feature a personal story alongside a descriptive analysis of the individual’s developmental configuration.

### Qualitative Analysis of “real life” personality developmental vignettes

Having derived a matrix for developmental authenticity the next paragraph will be dedicated to bridging theory and successful practical experience. Personality development and growth can be understood as an interactive and highly complex process. As many factors are involved, for example individual temperament and personality style dispositions, affect response in early childhood, as well as emotional attention and care, our general hypothesis is, that growth processes will not only individually vary but will especially be more successful through supportive environments. Experiences of helpful support as a direct and contingent response to stress show particularly positive effects on essential components of personal growth (Liesenfeld, 2018). The growth-dynamics presented within the following vignettes will illustrate different aspects of developmental cycles referring to our proposed authenticity matrix.

We selected certain client cases out of a large sample of a personality coaching practice, with three different growth-topics to be illustrated alongside the 4th C-Continuity criteria. All cases were anonymized and approved by the clients. The three clients underwent a pre-and mid-process-coaching assessment regarding their self-regulation competencies and are all considered examples for successful authenticity development. Through the following real-life anonymized cases we elucidate how the individual manages to authentically develop the relational dimensions of self-self and self-others by advancing from fragmented perception to holistic integration, specifically through helpful support during critical life phases.

### Ongoing Self-Assessment—a vignette for growing into “emotional freedom”

*Sally, a 48-year-old woman with a strong tendency for pleasing others and perfectionism, married at age 23. Her husband was an eloquent, value-oriented, and successful businessman. She considered herself happy and tried to please him whenever she could. Over the years, Sally and her husband had three children and two dogs. After the birth of the third child, Sally felt overwhelmed and suffered from long-term postnatal depression. Many of the things that made her happy before, suddenly seemed dull and frustrating. She questioned her life choices but felt too weak to change anything. She and her husband grew apart, lost intimacy over the course of ten years, merely connecting over their shared family roles. Sally felt dissociated and incapacitated*.

Ongoing Self-Assessment in a fragmented form is characterized by limited self-reflection. A person at a fragmented developmental level tends to experience new challenges as single, disconnected representations (Fischer, 1980). Further markers are a passive dissociation in different domains regarding context, task, and emotional state as well as a lack in awareness of own values and identity (likelihood of self-infiltration), (see Kuhl, 2001). In this case, Sally’s incongruence and disconnected representations displayed in sadness, distress, fear of intimacy exacerbated by the inability to stand up for her own needs. As unresolved negative emotions block the access to highly inferential systems (Kuhl, 2001), the modulation of pain into growth-oriented experience was impossible for her and thus developmental authenticity in this stage will be undermined through situation(state)-orientation (Baumann and Kuhl, 2021).

*When Sally’s first daughter graduated from high school, she started a coaching process and encouraged her mom to do the same, which Sally did. During this coaching process, Sally began to expand her personal territory. She started by examining what she likes and dislikes and evaluating her own role as wife and mother, based on underlying beliefs shaped through her biography. She had a strict father and developed a habit of pleasing in their relationship. She married young and directly continued this pleasing attitude with her husband. She realized that she had been absorbed by her husband’s needs. Sally’s personal growth process required her to increasingly observe her own behavior and attitude, specifically underlying beliefs that held her back from pursuing own needs earlier*.

In a partially integrated form Ongoing Self-Assessment is characterized by increasing self-complexity at the first level of integrating painful events. A person’s values and value-related behavior tend to be experienced consciously. Partial integration facilitates growth in certain domains (Fischer, 1980). Sally shows her partial integration process in connecting her perception of her relationship with her husband with the reflection on her upbringing and the role that her father played in her life to shape her individual perception of relating to others. By facing the painful aspects of those experiences, she was able to start integrating and thus widening her own ability to change her behavior. The decisive aspect of growth through her coaching process is the rising ability to self-confront negative emotions and painful insights and thus starting a transformation and integration process (see Kuhl, 2010). As nurturing and caring environments as well as adequate response during critical life incidents facilitate the process from fragmentation to integration (Liesenfeld, 2014; Baumann and Kuhl, 2021), the coaching environment provided the supportive framework.

*Sally’s coaching process revealed pain and frustration when she realized how far she went from her own needs. With every stage in the integration process, she felt how hard it was to learn to stand up for herself and build her own identity, growing out of the pleasing daughter with a fragmented view into a self-determined, integrated woman. She realized that she had to unlearn certain automatic responses and is still cultivating her own new identity. Meanwhile Sally and her husband managed to transform their marriage into a healthy, eye-to-eye, intimate relationship. Sally now runs her own consulting business, even consulting her husband’s firm on topics of personal development and leadership*.

Ongoing Self-Assessment mapped with holistic integration enables continuous evaluation and differentiation of the self. Holistically integrated people show openness and awareness for their own values, identity, and developmental stages within different contexts (Erikson, 1959; Fischer, 1980). They can hold space for their own and other’s strengths and weaknesses and show ambiguity tolerance. As there is not one fixed final level of holistic integration, this level describes the concept of a holistic-perception-ability that constitutes life-long personal growth. Erikson’s stages theory describes that within the process of holistic integration several developmental processes can align next to each other and across stages. As people climb from one stage to the next, not necessarily without full mastery, growth in one stage might allow for completing a growth-lack in a previous stage (Erikson, 1959).

*Sally managed to enter a personality growth level where she follows her own path and maintains a partnership-oriented, intimate relationship with her husband. She still occasionally encounters frustration while continuing to expand her horizon within various contexts of her personality*.

### Dynamic Change—a vignette for personal readjustment and growth

*George, an 82-year-old, healthy, and physically active man, recently lost his wife. He was married for nearly 60 years and loved his wife dearly. Previously, George had been an outgoing and content person with excellent communication skills. He had a fulfilling job and engaged regularly in sports and cultural activities. When his wife was diagnosed with cancer 15 years ago, he dynamically shifted his focus of happiness towards her well-being. In his own words, for him a happy day was when he was able to put a smile on his wife’s face. Throughout the stages of her illness, he was studying her condition, prepared himself for the regular doctors’ appointments and managed his wife’s life day to day. Although he continued with his regular sport activities as part of his self-care, he stopped going to the theater, skipped traveling, canceled personal invitations and within a few years he was socially isolated from his friends. He still considered himself as being happy and content, but from the outside view it was obvious that he had lost his spark, his eyes looked tired, and his attention and concentration seemed narrowly focused on his wife’s health condition*.

Dynamic Change on a fragmented developmental level is characterized by the tendency of a person to acquire knowledge and new skills as single, fragmented representations (Ayoub and Fischer, 2006). On that developmental level the individual is not able to re-evaluate their own self within the dynamic change and thus rather splits experiences that come with this change. George was so absorbed in his wife’s illness that he started to exclude feelings, experiences and opportunities that would have been meaningful for himself.

*George did not notice this shift, because he experienced positive stimulation organizing everything around the illness of his beloved wife. In fact, he over-engaged and continuously lost a significant part of his own self. When George’s wife finally needed to stay in the hospital, a certain shift occurred. George told his family that for the first time in many years he spent an entire day reading one of his favorite books and felt a certain relief. Simultaneously, he described feelings of shame, for his wife was not able to enjoy their recently newly built house while he was sitting in his armchair reading and contemplating*.

Partially integrated dynamic change processes are marked by a person’s partial ability to re-evaluate the self within dynamic change processes. On that level the individual is gaining competence in differentiating authentic from inauthentic aspects of the self and thus further processing single pieces of information in connection to (other) existing acquired information. This allows for increasingly complex competences and an integrative view through the development of parallel processing and integration networks (Kuhl, 2010). In George’s case it would not be correct to describe his behavior regarding his wife as inauthentic. He always had a nurturing personality and it felt authentic for him to help her, but he needed to detect for himself that the intensity and the exclusivity hardly left space for his personal life.

*When George’s wife passed away, he began to honestly confront himself. He started to visit places that they had enjoyed together, their favorite restaurant, their hiking trail, even the church they married in. Various memories and emotions came up that he felt deeply. One day he described an inner shift towards gratitude and grace, walking on their common life-trails. After a good amount of “self-confrontational training” as he put it, he could see the beauty in what he had with her over the loss. With this shift, he started to explore on his own, to see old friends and became part of a private hiking club that meets every other week*.

An individual that experiences Dynamic Change on a holistically integrated level shows an integrative competence to reevaluate the self within dynamic change processes. This reevaluation of authentic aspects of self goes alongside with the re-evaluation of non-authentic or suppressed self-aspects and thus reflects ambiguity tolerance where grief and loss can walk alongside with gratitude and grace. The ability to hold ambiguous stimuli within the extended network (extension memory) and thus to integrate painful events into the self-system in a self-directed manner (self-confrontational coping) indicates holistic integration and self-growth (Liesenfeld, 2018). After his wife passed away, George needed time to grieve and consequently to explore his own temporarily suppressed needs. As he confronted himself, he became increasingly able to hold the inner space to feel and accept the ambiguity of his new life situation without his wife.

### Breakage Points—a vignette for cultivating self-motivation and self-relaxation

*John, a 23-year-old student, was assessed as gifted with a significantly above average intelligence score in elementary school. He effortlessly solved all assigned tasks in school and was encouraged to skip one grade from 3rd to 5th grade. In 5th grade, his performance went down slightly but over the course of his school career he managed to finish with minimum effort at least with average grades. He started college at age 19 and soon realized that he could not get away with just being assessed as gifted. He had chosen a complex study field in environmental technology and physics. He needed to cope with a high volume of learning material, but as he never had learned the technique of studying, he was not able to prepare adequately and failed nearly all his classes. He kept this as a secret for more than 2 years and became disengaged and listless, unable to bear the burden any longer emotionally. Finally, he fell into a reactive depression, where he was hardly able to care for himself and isolated himself from his friends and loved ones*.

Breakage Points, like critical life incidents or specific growth cycles on a fragmented level of an individual’s perception lead to growth stagnation in certain areas and might even prevent personal growth, especially when the individual lacks helpful support during those critical times (Liesenfeld, 2014). John shows growth stagnation during the time he stayed isolated. He reported feelings of inner emptiness and an inability to act.

*After John told his parents what was going on, they took him home and supported him on many levels. They went to a physician, hired a coach, and tried to encourage him to restart and continue his studies. When they did not see progress, they tried to be strict and asked him to contribute to the finances for his college in order to motivate him. They role-modeled hard-working strategies, woke him up in the morning and offered joint learning hours. This helped John to slowly get into contact with a few of his suppressed feelings from the time when he skipped grades and felt isolated and ashamed, but it was not enough yet to get him to study properly on a regular basis*.

On a partially integrated level, Breakage Points lead to partial personal growth in certain domains. Helpful support, that is provided by caregivers, mentors, or other key relationship figures, will foster integration as this support allows for the negative affects to be confronted and for the self-system to open for integration (Liesenfeld, 2018). On this level the individual shows less suppression and avoidance of emotions (feelings), cognition and experiences (Fischer, 1980). Contingent support in stressful times buffers the related emotional experience and thus is one key factor for personal growth (Kuhl et al., 2015). John slowly started to show and verbalize emotions like listlessness, frustration, and anger.

*With the continuous support of his parents John underwent another intensive coaching program that was focused on the underlying brain functional dynamics of his behavior, respectively his suppressed emotions. His parents took part in family sessions as well to understand their son’s behavioral condition better. It became obvious that John—being very sensitive-was not only carrying his own suppressed feelings but on top of that he was carrying those of his parents, who were in a readjustment phase regarding their own career and partnership. When John’s father as the male role-model opened up to a deeper understanding of John’s lack of activity, John felt a significant relief. Both parents committed themselves to support John’s developmental path with a mixture of continuous coaching, mentoring and active learning support. From early on John held strong beliefs and a high attitude regarding performance and quality. This made it even harder to start his learning process. Whatever he did, he needed to start from scratch without the bonus of being gifted that had helped him, when he was younger. After several months John progressed significantly in his development. He started questioning his belief system and allowed himself to first take care of himself again, to be open to his own needs and wishes as well as starting to meet friends again. He now attends the choir in his hometown and joined a hockey club. It was and still is a bumpy road for him, but with progressive healthy developmental steps*.

Individuals that can holistically integrate Breakage Points, transform painful events and growth cycle effects into abstract mappings for authentic self-development (see Rogers, 1961; Fischer, 1980; Kuhl, 2010; Liesenfeld, 2018). This highest developmental and perception level is characterized by authentic personal growth through self-confrontation and integration of ambiguous stimuli within different contexts. As each stage of development can be completed at different times in life, growth is characterized by developing self-confrontational abilities and the ongoing integration of emotions (Erikson, 1959). John’s development gradually shows first signs of holistic integration patterns in certain contexts. Specifically in his private interactions with friends and his progressing self-care he shows new regulated behavior that underlines structural and dynamic adaptation via feedback processes (see Personality Dynamics Approach Carver and Scheier, 1998; see Dynamic Skill Theory Powers, 1973; Fischer, 1980). At John’s age (23) brain maturation is still in full progress, certain domains are in between growth cycles (Tamnes et al., 2010), so that it would be too early to expect a full range of holistic integration level in any domain.

*John is still in the process of confronting himself with painful events that he did not consider as valuable to talk about before. He can observe his own weak spots now without falling back into a depressive state and yet allowing himself negative feelings. He is starting to make plans and following through at least partially. His sport activities as well as his inner shifts are mirrored in his body posture. He carries his body more upright, can hold eye-contact and shows increasing self-confidence in the interaction with peers as well as with adults. Meanwhile, he even started a working group with other students to improve his performance*.

### Never-Ending-Story—recurring patterns and transgenerational imprinting

The main idea of this category is to highlight the infinity aspect of development. As brain and neural plasticity support continuous change in neural network connections over the lifespan, those changes are mainly provoked by the neurodynamical influence between our outer world and our inner world, i.e., the above mentioned self-self and self-other dynamics. Those dynamics seem to continuously reoccur and to be dependent on the ability to constantly self-assess and emotionally regulate to access the next higher developmental level.

Transgenerational patterns are observed, for example, in value and belief systems. Parenting beliefs (Erzinger and Steiger, 2014) or aggressive parenting styles (Conger et al., 2003) can lead children to later display the same beliefs, parenting styles, and behaviors they have observed. Various environmental influences (for example parenting behavior) have the capacity to modulate gene expression and consequently development and behavior (Haney et al., 1973; Verny and Kelly, 1982; Bakermans-Kranenburg and van Ijzendoorn, 2011; Liesenfeld, 2014; Vaiserman, 2014). The question arises, what kind of recurring patterns are bound to change? To what extent do those underlying transgenerational patterns such as values, beliefs, or trauma, that are not within the direct influence of the individual, either prevent or stimulate further development?

The Never-Ending-Story category can consist of all the three above mentioned process characteristics, described over the different developmental levels in the vignettes. It assumes, that in certain contexts or domains an individual might not be able to climb from one developmental level to the next, without having conscious control. The problem here is that not being able to access the next higher level developmental pathways will strengthen the existing pathways on a lower cognitive representation level and thus, creating even stronger networks for a certain maladaptation (more details in section 3, see Fischer, 1980).

Never-Ending-Story process characteristics on a fragmented developmental level lead to continuous growth-stagnation in specific domains over the course of development. Here the Never-Ending-Story is informed by recurring patterns of behavior and perception without conscious control. Those recurring patterns could source from maladaptation after traumatic experience or might be carried trans-generationally. Maladaptive behavior is defined as “behavior that interferes with an individual’s activities of daily living or ability to adjust to and participate in particular settings” (Gray, 2021, p. 1708). There is a broad spectrum of those behaviors, such as seeking reassurance to feel better about oneself, avoiding situations or thoughts, or excessive drinking and eating. One common link between those behaviors is the inability of people to endure negative affects and cope with it. Individuals feel threatened and overwhelmed by such experiences and try to resolve them with maladaptive behavior (Anestis et al., 2007).

These patterns of behavior are developed through learning experiences and may have once been useful but can reduce life satisfaction and in the worst-case lead to mental health issues (Buck, 2010). Maladaptive pathways such as maladaptive schemas and behaviors that are carried trans-generationally are characterized by a certain stability (Zeynel and Uzer, 2020). Referring to the example of Sally, her schema was self-deprecation, first formed by early experiences with her father and continued through her following close relationship. Due to the evolutionary process of conservation of species those maladaptive traits are more durable since on a lower cognitive level the avoidance of perceived danger and negative affect is stronger than confrontation and endurance of it (Ayoub and Fischer, 2006).

*Sally, in the above-mentioned vignette, had one specific domain where she was unable to leave a lower cognitive representation level. It was her close relationship-domain. As she was so used to being dependent and learned to please her overly strict father, it needed a breakage point for her (the danger of losing her husband and a serious depressive state) to be able to see the pattern and make new break-through choices within her regular self-assessment*.

On a partially integrated level Never-Ending-Story is marked by increasing conscious control over behavior and perception. Partial integration allows to detect and question some recurring patterns and facilitates adaptive change over the course of development. Never- Ending-Story on a partially integrated level enables the individual’s personal growth in certain domains.

*Within his developmental process John, the 23-year-old student shows recurring patterns that obviously were trans-generational traits of his family system. During the coaching process John became aware that one recurring pattern was an adapted focus on strict determined performance versus self-compatible, purpose-driven goal striving. As his parents served as role-models for highly disciplined performance, John did not learn to consciously connect his self-system with setting intentions to follow self-compatible goals. Additionally, emotions of shame and guilt connected to his non-performance kept the cycle never-ending. Starting to work on the underlying emotions helped John to increase his own awareness of cognitive and emotional learning blockage. While John was only able to concentrate on learning for 10 min in the past, he is now able to stay focused on learning for more than 2 h*.

Never-Ending-Story for an individual on a *holistically integrated level* manifests a growth mindset that allows for continuous transformation and personal growth. This level of development is marked by the ability to self-reflect on stagnating versus active growth patterns in self and others. The individual’s ability to reflect and change trans-generationally facilitates overarching personal growth and authentic self-development within family systems, learning and working environments, communities, and other contexts.

All three vignettes show the Never-Ending-Story on a holistically integrated aspect in reference to personal growth and continuous transformation. George, at the age of 82 was able to change his mind-set and gained a new quality of life satisfaction in accepting his personal loss next to the chance for a new chapter in his life.

Sally’s case shows the Never-Ending-Story in her continuous willingness to develop and learn from her past experiences. She started to have deep conversations with her daughter to reflect on her behavioral patterns as a mother and as a role-model specifically in close relationships. Sally meanwhile describes herself as a life-long-learner who still from time to time struggles in the relationship with her husband but can accept and cope with those situations of incongruence.

As John is only 23 years old and his brain maturation is still between growth cycles, a holistically integrated developmental level cannot be expected to the same extent compared to Sally and George. John’s Never-Ending-Story requires more experience and content to be filled with (but with continuous self-assessment, and a few more breakage points he will get there).

## Discussion

Highlighting the necessity of Inner Work in order to cope with increasing complexity and adverse contextual challenges, we came to address the dynamic developmental processes within an entity in context. In this article, we build on our recently published 4-C model (Dammann et al., 2021), where we highlighted congruence as the essence of authenticity by expanding the 3-C model of Lehman et al. (2019) with the concept of continuity. With this extension, we aim to dive deeper into the contradictory aspects within developmental processes that can show in non-uniform developmental patterns over different domains.

### Explicating continuity

A person will continuously go through the processes at every developmental level (see Table 2). During each phase of integration, a person’s domains of personality develop further and can be newly created, expanding the person’s sense of authenticity.

Assuming that a person on a fragmented level is not able to assess and evaluate relata, it is probable that this person has problems referring either to a self-self or self-world perspective and therefore experiences a limited sense of congruency. Throughout the developmental path, a person gains abilities in various process characteristics. This growth across the developmental levels leads to a more congruent self-self or self-world relationship.

In this paper, we have expanded our proposal of continuity as an important aspect of authenticity to include process characteristics (Ongoing Self-Assessment, Dynamic Change, Breakage Points, and Never-Ending Story) in combination with developmental levels (fragmentation, partial integration, and holistic integration), which are based on theories on development by Erikson (1959), Fischer (1980), and Kuhl (2001). We observe in our view of Developmental Authenticity a continuous process loop at each developmental level and across these levels. Individuals are continuously exploring and integrating new domains during their lifespan, which can be better understood through the framework of process characteristics and developmental levels. We believe holistic integration can never be fully obtained as a constant state but should be seen as a continuous, never-ending process within authenticity work (Peterson, 2005) and integration.

We have decided to incorporate theories focused on the developmental aspect of authenticity—the innate interactive dynamic that is authentic. While Kuhl’s (2001) PSI theory focuses on a functional approach, Fischer’s (1980) theory focuses on dynamic development, and Erikson (1959) emphasizes development, growth, and lifelong learning, which is not bound to a specific order over one’s lifespan. We refer to these theories in our approach to Developmental Authenticity with a focus on the individual.

In their individual development, children, unlike adults, are not capable of forming hierarchically organized and internally coherent constructs. This ability only matures in late adolescence to early adulthood. Development in early childhood understood as a complex change per time unit is not as comprehensive in any other stage. The very fact that this development occurs in a less linear and homogeneous manner, but rather domain-specifically, irregularly, and individually (cf. Kohlberg, 1969; Fischer, 1980; Piaget, 1983) makes a uniform picture of Developmental Authenticity difficult.

Our concept of Developmental Authenticity is a framework to understand individual development that otherwise cannot be compared since every individual is on their own journey through life. Therefore, we provided vignettes for a better understanding of this complex matrix of authentic personality development where our process characteristics and developmental levels serve as criteria in different contexts. Furthermore, these vignettes could serve as a practical guide for interventions and coaching with the development of individual branches of personality in mind. In addition, the vignettes emphasize the importance and influence of social networks and support to improve Self-Assessment, adapt to Dynamic Change, overcome Breakage Points, and accept the Never-Ending Story.

Our suggested matrix of Developmental Authenticity accounts for the often-neglected fact that development occurs within a body, which in turn acts in a physical and social context (Johnson, 1987; Noé, 2004). Current research increasingly supports this relationship and the interaction between person and context as an interwoven system (Clark, 1997; Lerner, 2005; Fischer and Bidell, 2006; Overton, 2006). The interaction between and mutual influencing of different psychological functions within every individual development demands a consideration of the integrative aspects of autonomous psychological processes (Ayoub and Fischer, 2006; Overton, 2007; Sneed et al., 2007; Witherington, 2007).

### Future implications

Through increasing complexity, the need for inner work becomes vital. Inner work consists of constant self-assessment, openness to dynamic change, and the ability to transform breakage points into personal growth.

For future work, we suggest our proposed matrix (Table 3) as a tool for coaching settings. The matrix may be used to document initial baseline assessment, visualize progress, and/or goal attainment status. It can be used in individual as well as educational settings.

Process characteristics and developmental levels may not be sufficient for a status assessment of a person because individual development is subject to change. As soon as there are more than two parameters, it becomes difficult to statistically analyze personality development. Instead, the behavior and self-perception of a person need to be observed within the parameters of the suggested matrix of Developmental Authenticity to enable a holistic view. Moreover, our approach to Developmental Authenticity requires an individualized application for every person. The vignettes are examples of how differentiated personal development journeys can be. This framework might allow us to develop additional and more conducive instruments.

Our approach to mapping various configurations of developmental process characteristics and developmental levels proposes to use this authenticity matrix to differentiate by domain-specific personal growth rather than simply assessing individuals at an overall developmental maturity level.

## Data availability statement

The original contributions presented in the study are included in the article/supplementary material, further inquiries can be directed to the corresponding author.

## Ethics statement

Ethical approval was not required for the study involving humans in accordance with the local legislation and institutional requirements. Written informed consent to participate in this study was not required from the participants or the participants’ legal guardians/next of kin in accordance with the national legislation and the institutional requirements. Written informed consent was obtained from the individual(s) and/or minor(s)’ legal guardian/next of kin for the publication of any potentially identifiable images or data included in this article.

## Author contributions

KL is the primary contributor of the concept, the proposed theoretical approach and the vignettes. All authors contributed to the research and writing and approved the manuscript for publication.
